# Saikosaponin-d Inhibits the Hepatoma Cells and Enhances Chemosensitivity Through SENP5-Dependent Inhibition of Gli1 SUMOylation Under Hypoxia

**DOI:** 10.3389/fphar.2019.01039

**Published:** 2019-09-20

**Authors:** Chun-Yan Zhang, Zhong-Min Jiang, Xiao-Fang Ma, Yue Li, Xiao-Zhi Liu, Li-Li Li, Wen-Han Wu, Tao Wang

**Affiliations:** ^1^Department of Pharmacy, Tianjin Binhai New Area Hospital of Traditional Chinese Medicine, Tianjin, China; ^2^Tianjin State Key Laboratory of Modern Chinese Medicine, Tianjin University of Traditional Chinese Medicine, Tianjin, China; ^3^Department of Pathology, The Fifth Central Hospital of Tianjin, Tianjin, China; ^4^Central Laboratory, The Fifth Central Hospital of Tianjin, Tianjin, China; ^5^Department of Bone and Soft Tissue Tumors, Tianjin Medical University Cancer Institute and Hospital, Tianjin, China; ^6^National Clinical Research Center for Cancer, Tianjin Medical University Cancer Institute and Hospital, Tianjin, China; ^7^Key Laboratory of Cancer Prevention and Therapy, Tianjin Medical University Cancer Institute and Hospital, Tianjin, China; ^8^Department of General Surgery, Peking University First Hospital, Beijing, China; ^9^Department of General Surgery, The Fifth Central Hospital of Tianjin, Tianjin, China

**Keywords:** Saikosaponin-d, hepatocellular carcinoma, HSVtk/GCV, Gli1, SUMOylation, SENP5, Hep3B cells, hypoxia

## Abstract

Chemosensitivity is one of the key factors affecting the therapeutic effect on cancer, but the clinical application of corresponding drugs is rare. Hypoxia, a common feature of many solid tumors, including hepatocellular carcinoma (HCC), has been associated with resistance to chemotherapy in part through the activation of the Sonic Hedgehog (SHh) pathway. Hypoxia has also been associated with the increased SUMOylation of multiple proteins, including GLI family proteins, which are key mediators of SHh signaling, and has become a promising target to develop drug-resistant drugs for cancer treatment. However, there are few target drugs to abrogate chemotherapy resistance. Saikosaponin-d (Ssd), one of the main bioactive components of *Radix bupleuri*, has been reported to exert multiple biological effects, including anticancer activity. Here, we first found that Ssd inhibits the malignant phenotype of HCC cells while increasing their sensitivity to the herpes simplex virus thymidine kinase/ganciclovir (HSVtk/GCV) drug system under hypoxia *in vitro* and *in vivo*. Furthermore, we had explored that GLI family activation and extensive protein SUMOylation were characteristics of HCC cells, and hypoxia could activate the SHh pathway and promote epithelial-mesenchymal transition (EMT), invasion, and chemosensitivity in HCC cells. SUMOylation is required for hypoxia-dependent activation of GLI proteins. Finally, we found that Ssd could reverse the effects promoted by hypoxia, specifically active sentrin/small ubiquitin-like modifier (SUMO)-specific protease 5 (SENP5), a SUMO-specific protease, in a time- and dose-dependent manner while inhibiting the expression of SUMO1 and GLI proteins. Together, these findings confirm the important role of Ssd in the chemoresistance of liver cancer, provide some data support for further understanding the molecular mechanisms of Ssd inhibition of malignant transformation of HCC cells, and provide a new perspective for the application of traditional Chinese medicine in the chemical resistance of liver cancer.

## Introduction

Hepatocellular carcinoma (HCC) is the leading cause of cancer-related deaths worldwide and is largely asymptomatic but a highly aggressive disease (Siegel et al., 2018; Marino et al., 2019). Chemotherapy is the main method commonly used in the treatment of cancer, and it will become the most effective and promising method for the treatment of malignant tumors (Moriguchi et al., 2016; Ikeda et al., 2018). However, the drug resistance of tumor cells seriously affects the clinical chemotherapy effect of the drug (Liang and Aszalos, 2006). According to the American Cancer Society, more than 90% of cancer patients die from different degrees of tumor cell resistance. Therefore, enhancing the sensitivity of chemotherapeutic drugs has become a new research hot spot.

Some studies indicate that there are many reasons for drug resistance, including hypoxia, tumor suppressor gene mutation, and cell membrane transporter expression (Szakacs et al., 2006), which cause tumor cells to mutate, structure, and function to evolve and are not sensitive to drugs, then ultimately lead to failure of chemotherapy. At present, researchers are devoting themselves to improving the sensitivity of chemotherapy drugs and overcoming the drug resistance of tumor cells. But this problem has not been fundamentally solved, the clinical effects of some drugs are still not satisfactory.

Natural products from traditional Chinese medicine with highly diverse bioactivities and functions play a dominant role in the chemosensitivity in hepatoma. In recent years, many natural products have been found to reverse the chemotherapeutic resistance of cancer cells and play an increasingly important role in this regard, such as dauricine (Li et al., 2018), quercetin (Chen et al., 2018), and so on. However, their mechanism of action is still far from clarified and needs further study.

Saikosaponin-d (Ssd), a major bioactive triterpenoid saponin from *Radix bupleuri*, has been shown to demonstrate anti-inflammatory (Lu et al., 2012), anticancer (Zhong et al., 2016), and antioxidative (Zhang et al., 2014) properties. Recent studies have demonstrated that Ssd can promote radiosensitivity in HCC cells (Wang et al., 2014). However, the potential for Ssd to alter the efficacy of chemotherapy and its specific mechanism of action remain largely unexplored.

In the current study, we have found that Ssd suppresses proliferation and migration of HCC cells and enhances the sensitivity of these cells to herpes simplex virus thymidine kinase/ganciclovir (HSVtk/GCV) treatment in hypoxic conditions *in vitro* and *in vivo*. Furthermore, we revealed that Ssd specifically activates sentrin/small ubiquitin-like modifier (SUMO)-specific protease 5 (SENP5), strongly inhibiting SUMO1 and Gli1 and reversing the effects promoted by hypoxia. Our findings proved the important role of Ssd in the chemoresistance of liver cancer and will provide a possible molecular mechanism for the antitumor properties of traditional Chinese medicine.

## Materials and Methods

### Drugs

Ssd (SS8010, ≥98% purity) was purchased from Beijing Solarbio Science & Technology Co., Ltd. Spectomycin B1 (SB1, S4014, ≥98% purity) was obtained from Sigma (Sigma-Aldrich, St. Louis).

### Human Tissue Specimens

Fresh surgical specimens (tumor and matched adjacent nontumor tissues) were collected from 10 patients with HCC who underwent surgical resection at the Fifth Central Hospital of Tianjin (Tianjin, China) between January and December 2017. The initial diagnosis of all frozen samples was conducted by a senior pathologist. Subsequent paraffin-embedded sections were reexamined by a different pathologist to confirm the initial diagnosis. The study was approved by the Biomedical Research Ethics Committee of the Fifth Central Hospital of Tianjin, and all patients provided informed consent.

### Cell Lines and Cell Culture

Hep3B cells were obtained from the American Type Culture Collection (ATCC, Maryland, USA) and were confirmed negative for mycoplasma contamination. The cells were cultured in Dulbecco’s modified Eagle’s medium (DMEM) supplemented with 10% fetal bovine serum (FBS), 100 U/ml penicillin, and 100 µg/ml streptomycin (all from Gibco; Thermo Fisher Scientific, Inc., Waltham, MA, US) at 37ºC in a humidified atmosphere containing 5% CO_2_. For hypoxia treatment, the cells were cultured in an atmosphere of 3% oxygen for 24 h. Cells cultured in normoxic conditions (21% oxygen) were used as controls.

### Cell Proliferation Assay

Cell proliferation was evaluated using the Cell Counting Kit-8 (CCK-8; Yeasen, Shanghai, China). The cells were cultured in 96-well plates for 24 h in normal medium. For dose evaluation assays, the culture medium was then changed for fresh media containing a range of Ssd concentrations (0, 2, 4, 6, 10, and 15 µM), and the cells were then cultured for a further 48 h. To determine changes in cell proliferation, 10 µl of CCK-8 (5 mg/ml) was added to each well, and the cells were then incubated for 1 h before measuring absorbance at 450 nm using a microplate reader (Bio-Rad, Hercules, CA, USA). Finally, absorbance values were used to derive the cell number from a standard curve.

### Western Blotting

Proteins were extracted from tissues or cultured cells using RIPA buffer (Solarbio, Beijing, China) supplemented with 1 mM phenylmethanesulfonyl fluoride and 20 mM N-ethylmaleimide. Protein concentrations were measured using the BCA Protein Quantification Kit (Thermo Fisher Scientific, Inc.). Total proteins (100 µg per lane) were then resolved on 10% SDS-PAGE gels and subsequently transferred onto polyvinylidene fluoride membranes (EMD Millipore, Inc.). Membranes were then probed with antibodies for specific target proteins. Information on all antibodies is shown in **Table 1**. Data were evaluated by image analysis software (ImageJ version 1.48; National Institutes of Health, Bethesda, MD, USA).

**Table 1:** The information of all related antibodies in this study.

Antibody	Gli1	Gli2	SUMO2/3	SHH	PTCH1	Smoothened	Vimentin	E-Cadherin	β-Actin
Company	Abcam	Abcam	Abcam	Abcam	Abcam	Abcam	CST	BD	Abcam
Code	ab151796	ab187386	ab3742	ab53281	ab39266	ab38686	#5741	610404	ab8227
Application	WB/IHC	WB/IHC	WB	WB	WB	WB	WB	WB	WB
Dilution	1:1,000/1:500	1:1,000/1:500	1:1,000	1:2,000	1:1,000	1:1,000	1:1,000	1:500	1:1,000
**Antibody**	**Gli3**	**SUMO1**	**Ubc9**	**SENP1**	**SENP2**	**SENP3**	**SENP5**	**SENP6**	**SENP7**
Company	Abcam	Abcam	Abcam	Abcam	Abcam	Abcam	Abcam	Abcam	Abcam
Code	ab6050	ab11672	ab75854	ab108981	ab58418	ab71677	ab183477	ab57239	ab58422
Application	WB/IHC	WB/IHC	WB	WB	WB	WB	WB	WB	WB
Dilution	1:1,000/1:500	1:2,000/1:400	1:2,000	1:2,000	1:800	1:200	1:2,000	1:500	1:800

### Wound Healing Assay

Hep3B cells (0.25 × 10^6^/well) were seeded into six-well plates and cultured until a cell monolayer had established. Scratch wounds were then gently made in the monolayer cultures using a 200 µl pipette tip. Then, cells were treated with Ssd in normoxic or hypoxic conditions. An inverted microscope (Olympus, Japan) was used to acquire digital images of wounds at 0 h and 24 h postscratching, and ImageJ was then used to measure the wound width. Wound width values were subsequently used to quantify cell migration.

### Invasion Assay

To evaluate cell invasion, cells (1 × 10^4^ in 100 µl serum-free medium) were seeded into the upper well of transwell chambers, and medium containing 10% FBS was placed in the lower well. Prior to cell seeding, transwell chamber membranes were precoated with Matrigel (BD Biosciences) for 30 min at 37ºC. The cells were then incubated for 48 h at 37ºC in a 5% CO_2_ humidified atmosphere. The medium was removed from the upper chamber, and the membranes then stained with crystal violet for 10 min. The inserts were then imaged using an inverted microscope, and cells that had invaded through the Matrigel were counted using ImageJ.

### Apoptosis Assays

Hep3B cells were treated with HSVtk/GCV in normoxic or hypoxic culture conditions for 24 h and then assayed for apoptotic cell death using a TdT-mediated dUTP nick end labeling (TUNEL) assay kit (Roche Diagnostics). Briefly, cells were harvested and fixed in 4% paraformaldehyde, stained using TUNEL reaction mixture, and then incubated in the dark for 1 h at 37ºC. Cell nuclei were then counterstained with DAPI (Zhongshan Goldenbridge, Beijing, China), and cell staining subsequently analyzed using a confocal fluorescence microscope (Carl Zeiss AG, Germany).

Cell apoptosis was also assessed by flow cytometry. Cells cultured under normoxic or hypoxic conditions for 24 h were harvested by trypsinization and then stained using the Annexin V Apoptosis Detection Kit (BD Biosciences) according to manufacturer’s instructions. The labeled cells were then analyzed by flow cytometry to detect early and late apoptotic cells.

### Immunofluorescence

Immunofluorescence (IF) was performed on HCC cells cultured *in vitro* to evaluate changes in protein expression and subcellular localization. Cells cultured on glass coverslips were fixed with 4% paraformaldehyde, washed with PBS, permeabilized in 0.2% Triton X-100 for 10 min, and then blocked in 5% bovine serum albumin for 1 h. Next, the cells were incubated with primary antibody against Gli1-3 overnight at 4ºC, washed three times with PBS, and then incubated with Alexa Fluor 488-conjugated anti-rabbit IgG H&L (ab150080, Abcam; 1:500) for 1 h in the dark. Cell nuclei were counterstained with DAPI for 20 min before assessing protein expression and subcellular localization using a confocal microscope.

### Hematoxylin and Eosin Staining and Immunohistochemistry

Tissue sections were deparaffinized in xylene and rehydrated with degraded alcohol. Samples were then either subjected to hematoxylin and eosin (H&E) staining or further processed for immunohistochemistry (IHC). For IHC, samples were blocked in PBS (pH 7.4) containing 5% bovine serum albumin (BSA) and then incubated with primary antibodies overnight at 4ºC. Primary antibodies and their dilutions for IHC were shown in **Table 1**. Immunoreactivity was detected using a universal two-step detection kit (PV-8000, Zhongshan Goldenbridge, Beijing, China).

### Analysis of Cellular Lactate Dehydrogenase Levels by ELISA

HSVtk/Hep3B cells cultured in normoxic or hypoxic conditions were incubated with Ssd or GCV either alone or in combination for 24 h. The cells were then collected and subjected to low-temperature sonication, followed by centrifugation at 12,000 rpm for 10 min. The supernatant was collected, and cellular levels of lactate dehydrogenase (LDH) subsequently determined using the enzyme-linked immunosorbent assay (ELISA)-based LDH Activity Assay Kit (Yuanmu Biotechnology Co., Ltd., Shanghai, China), according to the manufacturer’s instructions.

### Experimental Mouse Model

Four-week-old BALB/c female athymic nude mice (Beijing Vital River Laboratory Animal Technology Co., Ltd.) were inoculated by subcutaneous injection of HSVtk/Hep3B cells (5 × 10^6^ in 0.1 ml PBS). When tumor volume reached 100 mm^3^, mice were randomly assigned into four experimental groups (n = 9 per group) that subsequently received vehicle, Ssd (10 mg/ kg), GCV (15 mg/kg), or combined Ssd and GCV therapy *via* intraperitoneal injection every other day for 33 days. Tumor volume and body weight were recorded every 3 days. This experiment was performed according to the regulations and guidelines approved by the Animal Ethics Committee of the Fifth Central Hospital of Tianjin (Tianjin, China).

### Statistical Analysis

All data are presented as means ± SD and were analyzed using GraphPad Prism 6 software (San Diego, CA). One-way analysis of variance (ANOVA) or the unpaired Student’s *t*-test was used to evaluate the significance of differences among treatment groups, as appropriate. A value of *P* < 0.05 was considered to be statistically significant. All experiments were repeated at least three times.

## Results

### GLI Family Activation and Extensive Protein Sumoylation Are Characteristics of HCC Cells

**Figure 1:**
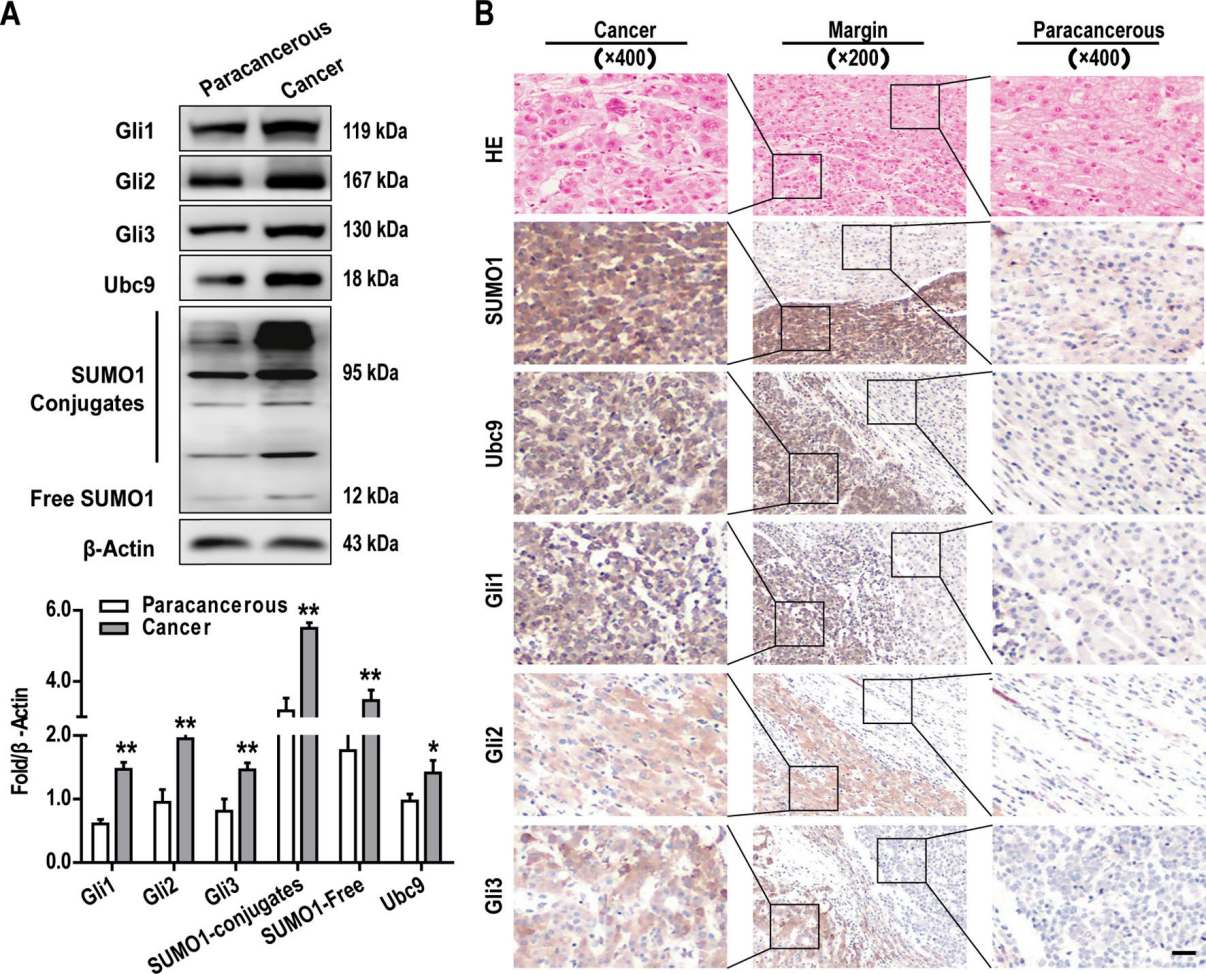
Elevated GLI family proteins expression and extensive protein SUMO modification are characteristics of hepatocellular carcinoma (HCC) cells *in vivo*. Gli1-3, SUMO1, and Ubc9 protein expressions were examined by **(A)** western blotting and **(B)** immunohistochemistry (IHC) (scale bar, 50 µm) in tumors and adjacent normal tissues. Data are shown as means ± SD (n = 3); *P < 0.05 and **P < 0.01 when compared with adjacent normal tissues (Student’s t test).

To explore the role of the GLI family proteins (include Gli1, Gli2, Gli3) in the onset and development of HCC, we examine tumor and matched adjacent normal tissue samples from 10 patients with HCC. GLI protein expression in tissue samples were examined by western blotting and IHC. In 8 of the 10 cases, both methods revealed that the expression of all GLI proteins was significantly increased in HCC tissues when compared with normal adjacent tissues (**Figures 1A, B**), a finding which is consistent with those for other cancer types, including gastric, colorectal, lung, breast, and pancreatic cancer (Kasper et al., 2006; Lauth and Toftgard, 2007; Song et al., 2017; Didiasova et al., 2018).

It has been reported that SUMO modification is involved in the formation and progression of tumors (Bialik and Wozniak, 2017). It is also known that GLI proteins can be modified by SUMO, which influences the transcriptional activity of these proteins (Ma et al., 2016; Hoard et al., 2018). Therefore, we explored the expression of important targets of the SUMO pathway (SUMO1 and Ubc9) in HCC tumor tissues with matched normal adjacent tissues. Western blotting analysis demonstrated that SUMO1 and Ubc9 were both highly expressed in HCC tissues (**Figure 1A**), and it can be seen that some bands bound to SUMO1, which may be a large amount of protein SUMOylation. A similar tendency was also observed when the expression of these proteins was examined by IHC (**Figure 1B**). Importantly, the IHC analysis revealed that all of the above proteins localized predominantly to the cytoplasm in HCC cells (**Figure 1B**) and that their expression was more than two-fold greater than that observed in the cells of adjacent noncancerous tissues.

### Hypoxia Activates the SHh Pathway and Promotes Epithelial-Mesenchymal Transition, Invasion, and Chemosensitivity in Hep3B Cells

Hypoxia is known to directly regulate the proliferation and invasion of cancer cells and also to promote the epithelial-mesenchymal transition (EMT), an important driver of metastasis in many cancers (Mao et al., 2016; Amawi et al., 2017; Joseph et al., 2018).

**Figure 2:**
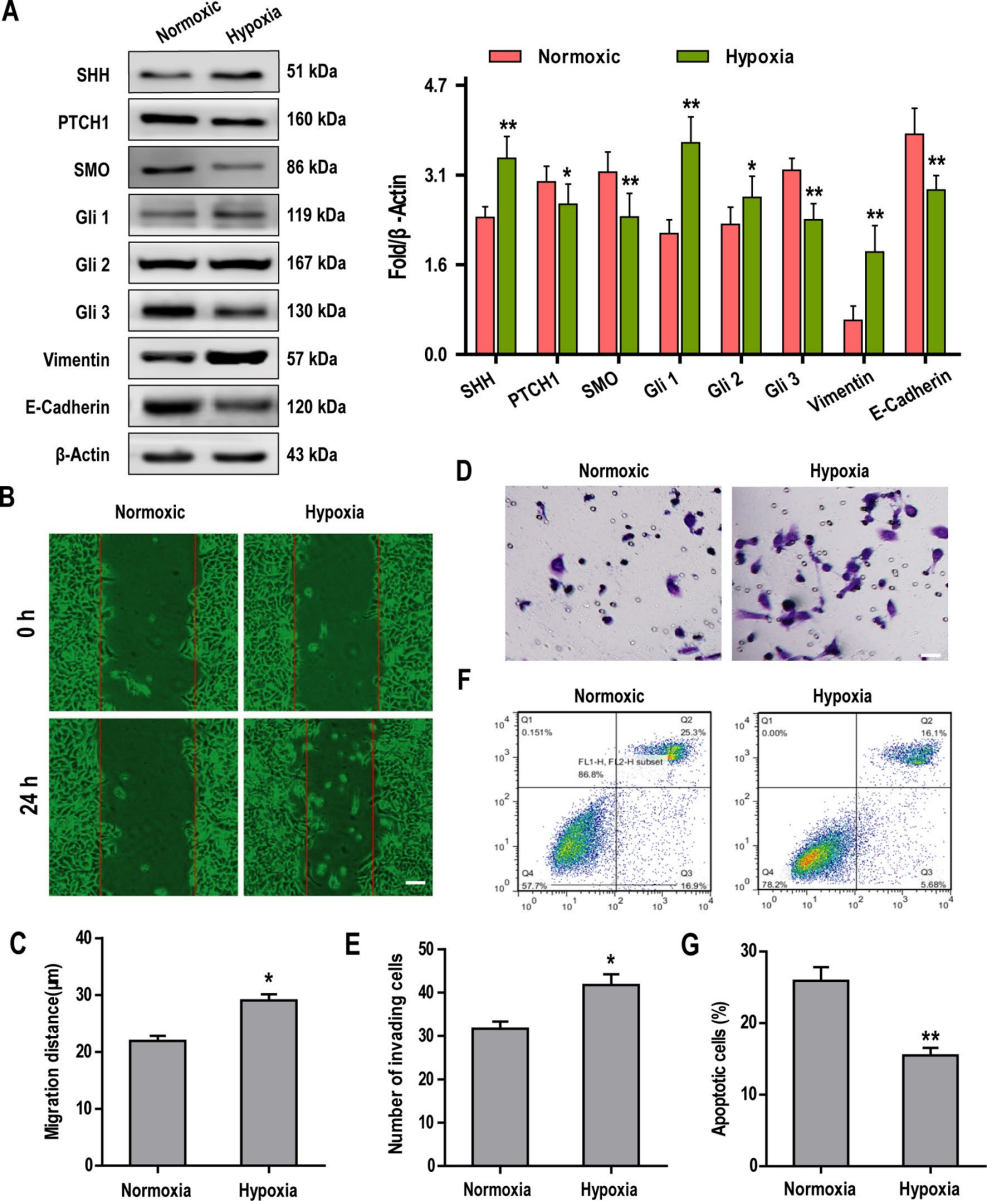
Hypoxia activates the Sonic Hedgehog (SHh) pathway and promotes epithelial-mesenchymal transition (EMT), invasion, and chemosensitivity in Hep3B cells. **(A)** The expression of SHh pathway and key EMT-associated proteins were examined by western blotting. **(B**, **C)** Hep3B cell migration under normoxic and hypoxic conditions was analyzed over 24 h using a wound healing assay (scale bar, 50 µm). **(D**, **E)** Hep3B cell invasion was examined by transwell assay (scale bar, 50 µm). **(F**, **G)** Cell apoptosis was examined by flow cytometry. Data are shown as means ± SD (n = 3); *P < 0.05 and **P < 0.01 when compared with the control (Student’s t test).

Recently, Sonic Hedgehog (SHh)-GLI signaling has been shown to play a key role in the induction of EMT (Yue et al., 2014). To investigate the cross-talk between the SHh pathway and hypoxia-dependent induction of EMT in Hep3B cells, we examined the expression of key proteins associated with both of these pathways. Our data showed that hypoxia promoted the expression of SHH, Gli1, Gli2, and vimentin but significantly suppressed the expression of PTCH1, Smoothened (SMO), Gli3, and E-Cadherin in Hep3B cells when compared with normoxic controls (**Figure 2A**). Further experiments revealed that Hep3B cell migration and invasion were significantly increased under conditions of hypoxia (**Figures 2B–E**). In contrast, flow cytometry analysis revealed that the apoptosis of Hep3B cells treated with HSVtk/GCV system decreased significantly when cells were cultured under hypoxia (cf, 25.3% for normoxic controls vs. 16.1% for hypoxic cultures; **Figures 2F, G**).

### Ssd Suppresses the Malignant Phenotype in Hep3B Cells and Increases Sensitivity to Chemotherapy Under Hypoxic Conditions

**Figure 3:**
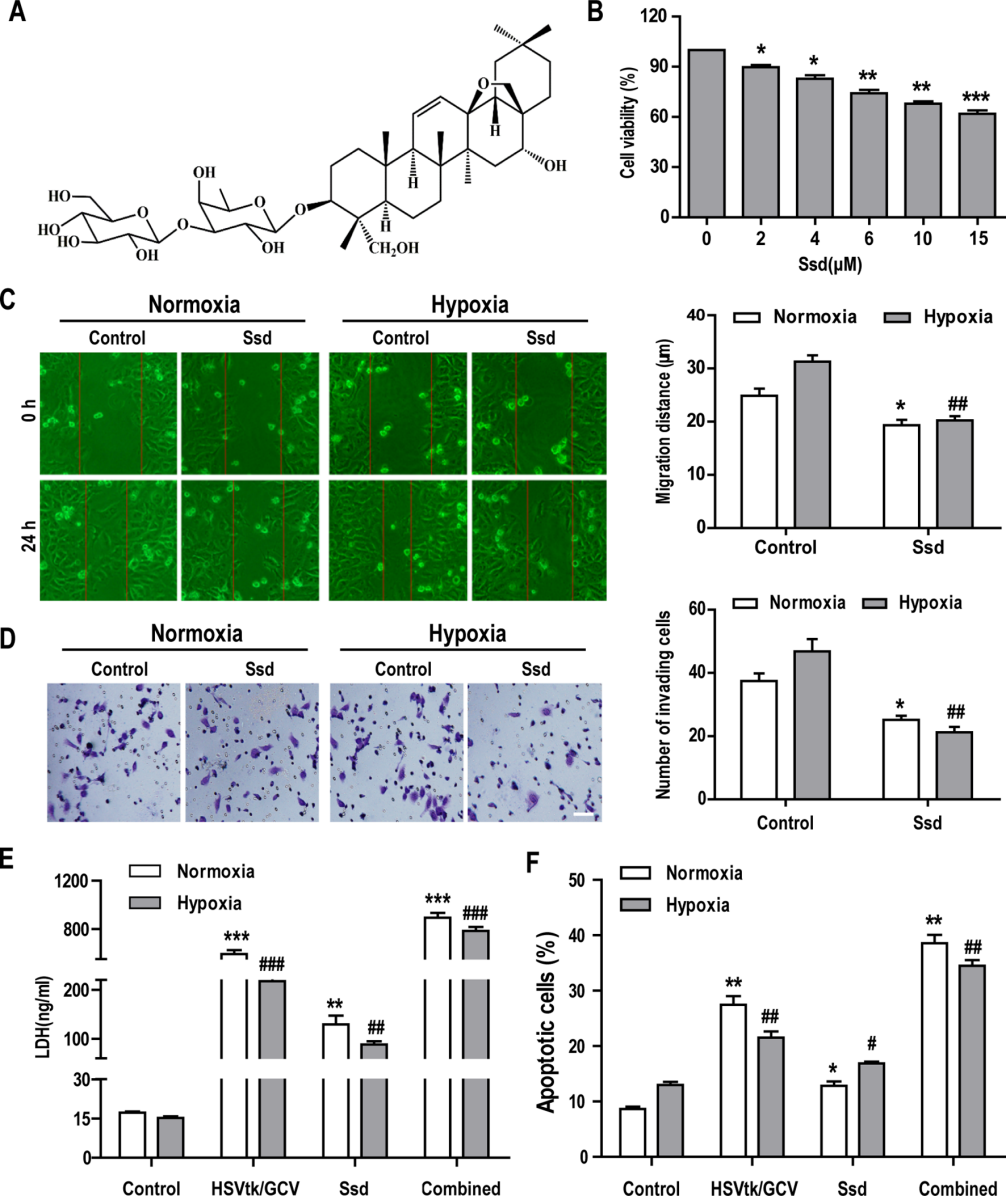
Saikosaponin-d (Ssd) markedly inhibits the malignant phenotype in Hep3B cells and increases their sensitivity to chemotherapy under hypoxic conditions. **(A)** Chemical structure of Ssd. **(B)** The viability of Hep3B cells treated with different concentrations of Ssd for 48 h was examined using the CCK-8 assay. **(C)** The effects of Ssd (6 µM) on the migration of Hep3B cells under normoxic and hypoxic conditions were examined over 24 h by wound healing assay (scale bar, 50 µm). **(D)** The effects of Ssd on the invasive potential of Hep3B cells were examined by transwell assay under normoxic and hypoxic conditions (scale bar, 50 µm). **(E)** Lactate dehydrogenase (LDH) expression in Hep3B cells was measured by enzyme-linked immunosorbent assay (ELISA) following exposure of cells to the indicated treatments. **(F)** Hep3B cell apoptosis was examined by flow cytometry. Data are shown as means ± SD (n = 3); *P < 0.05, *P < 0.01, and *P < 0.001 when compared with the normoxia control; *^#^* *P* < 0.05, *^##^* *P* < 0.01, and *^###^* *P* < 0.001 when compared with the hypoxia control (Student’s t test).

The chemical structure of Ssd is shown in **Figure 3A**. The CCK-8 cell viability assay was used to assess the cytotoxic effects of increasing concentrations of Ssd toward HepG3B cells over a 48 h period. The results in **Figure 3B** show that Ssd had a dose-dependent inhibitory effect on Hep3B cell viability. Hypoxia is a common feature of solid tumors and is thought to play a major role in promoting tumor cell invasion, metastasis, resistance to radiotherapy and chemotherapy, and recurrence during tumor development (Manoochehri Khoshinani et al., 2016; Krzykawska-Serda et al., 2017).

To evaluate the effects of Ssd on the hypoxia-associated malignant phenotype, we treated Hep3B cells with Ssd under normoxic or hypoxic conditions and subsequently analyzed various aspects of malignant cell behavior. First, cell migration and invasion were measured *in vitro* using wound healing and transwell assays, respectively. In wound healing assays, in which cell migration was evaluated over a 24 h period, the relative wound gap was larger for the Ssd treatment group (31.28 ± 3.67 µm) than for the control group (19.32 ± 3.28 µm) in both normoxic and hypoxic culture conditions (**Figure 3C**). Importantly, this Ssd-dependent inhibition of cell migration was more pronounced under hypoxia. Consistent with these findings, transwell assays revealed that Ssd also had a suppressive effect on the hypoxia-dependent invasion of Hep3B cells (**Figure 3D**). Ssd suppressed invasion under both normoxic and hypoxic conditions but had a greater impact on hypoxia treatment, resulting in a greater than two-fold reduction in the number of invading cells. To explore the potential sensitizing effects of Ssd to chemotherapy in Hep3B cells, we used the established herpes simplex virus thymidine kinase/ganciclovir (HSVtk/GCV) therapeutic system, which has been shown to be effective for most cancer cell types (Xiao et al., 2019). The results from the LDH activity and apoptosis assays confirmed the increase in cell death following the treatment of HSVtk/Hep3B cells with Ssd or GCV and, importantly, revealed that the combined effect of these treatments was significantly greater than when either was used alone (**Figures 3E, F**).

### Sumoylation Is Required for Hypoxia-Dependent Activation of GLI Proteins

**Figure 4:**
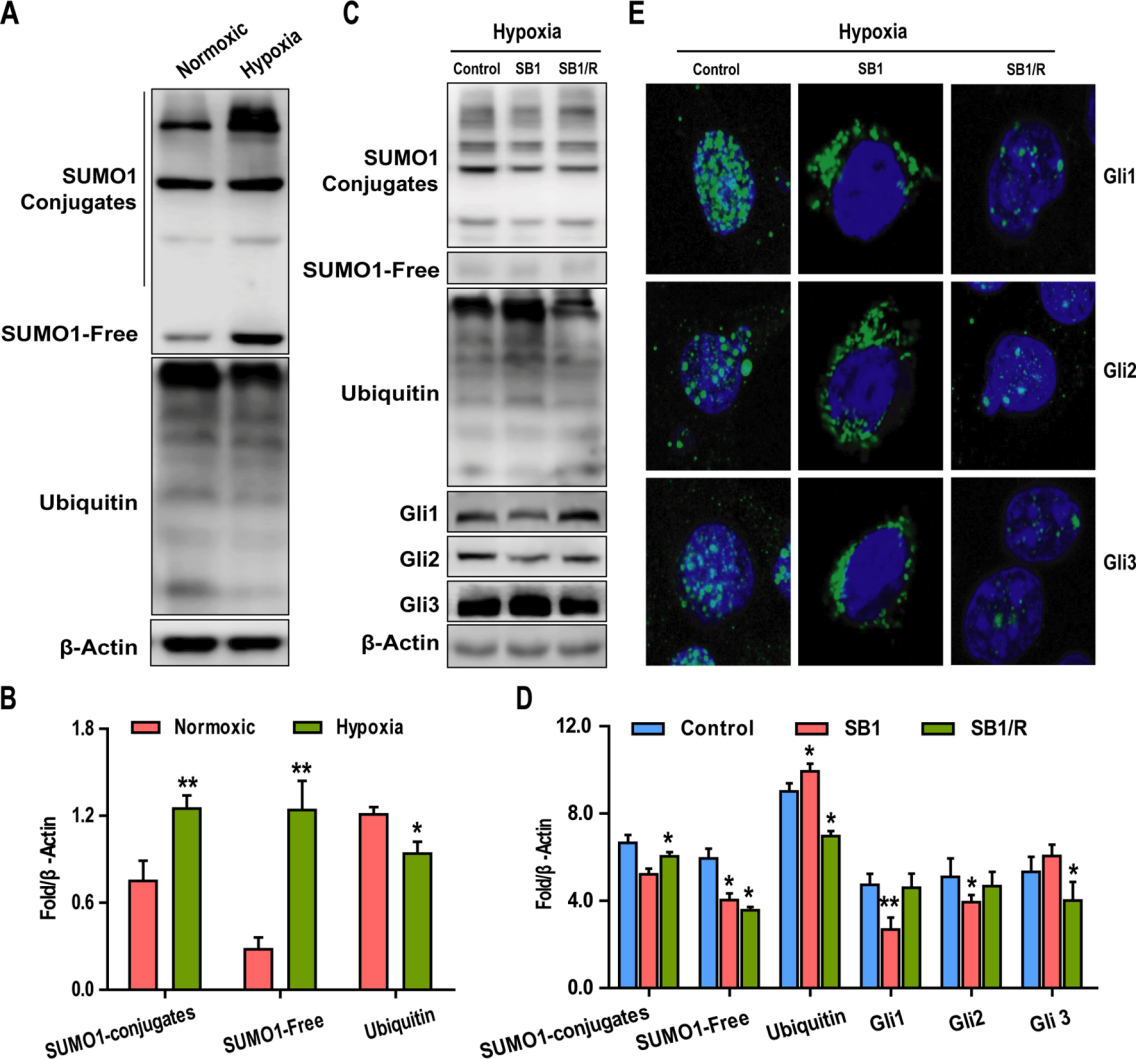
SUMOylation is required for the hypoxia-dependent activation of GLI proteins. **(A**, **B)** The expressions of SUMO1, Ubc9, and Ubiquitin under normoxic and hypoxic conditions were examined by western blotting. **(C**, **D)** Western blotting analysis of SUMO1, Ubc9, Ubiquitin, and Gli1-3 expression in Hep3B cells subjected to the indicated treatments. **(E)** The subcellular localization of GLI proteins in Hep3B cells treated as in **C** was examined by immunofluorescence (IF) (scale bar, 50 µm). DAPI was used to stain cell nuclei. Data are shown as means ± SD (n = 3); *P < 0.05 and **P < 0.01 when compared with control (Student’s t test).

Although we have shown that hypoxia can induce activation of the SHh pathway, the role of SUMOylation in this process remains to be determined. To this end, we first examined the effects of hypoxia on SUMO1 and Ubiquitin (CST, #3936,1:1000) expression. The results revealed that levels of SUMO1 (both conjugates and free forms of the protein) in Hep3B cells were significantly higher under hypoxic conditions than normoxic conditions, whereas the expression of Ubiquitin was reduced under hypoxic conditions (**Figures 4A, B**). To investigate the possible role of SUMOylation in the hypoxia-dependent activation of SHh signaling, Hep3B cells were treated with spectomycin B1 (SB1), a novel SUMOylation inhibitor. Hep3B cells were treated with SB1 (20 µM) for 24 h or with SB1 for 12 h followed by a 12 h recovery (SB1/R) under conditions of hypoxia. Untreated cells cultured under hypoxic conditions served as the control. After the 24 h experimental period, the effects of inhibition of SUMOylation on GLI members were determined by western blotting and IF. As shown in **Figures 4C, D**, SB1 promotes the downregulation of SUMO1, Gli1, and Gli2 and the upregulation of Ubiquitin and Gli3 in Hep3B cells whereas removal of SB1 (SB1/R) reversed the effect. IF was also performed to determine changes in the intensity and subcellular localization of Gli1-3 following these treatments. The IF analysis demonstrated that all GLI family proteins had a predominantly nucleus distribution under hypoxia conditions, whereas a significant shift to the cytoplasmic was observed upon SB1 treatment (**Figure 4E**). This nuclear relocalization phenotype was particularly apparent for Gli1. Furthermore, nuclear relocalization of GLI proteins was reversed once SB1 was removed from the culture medium. These data suggest that SUMOylation is required for hypoxia-induced activation of GLI proteins.

### Ssd May Inhibit the Sumoylation of Gli1 by Specifically Upregulating the Expression of SENP5

**Figure 5:**
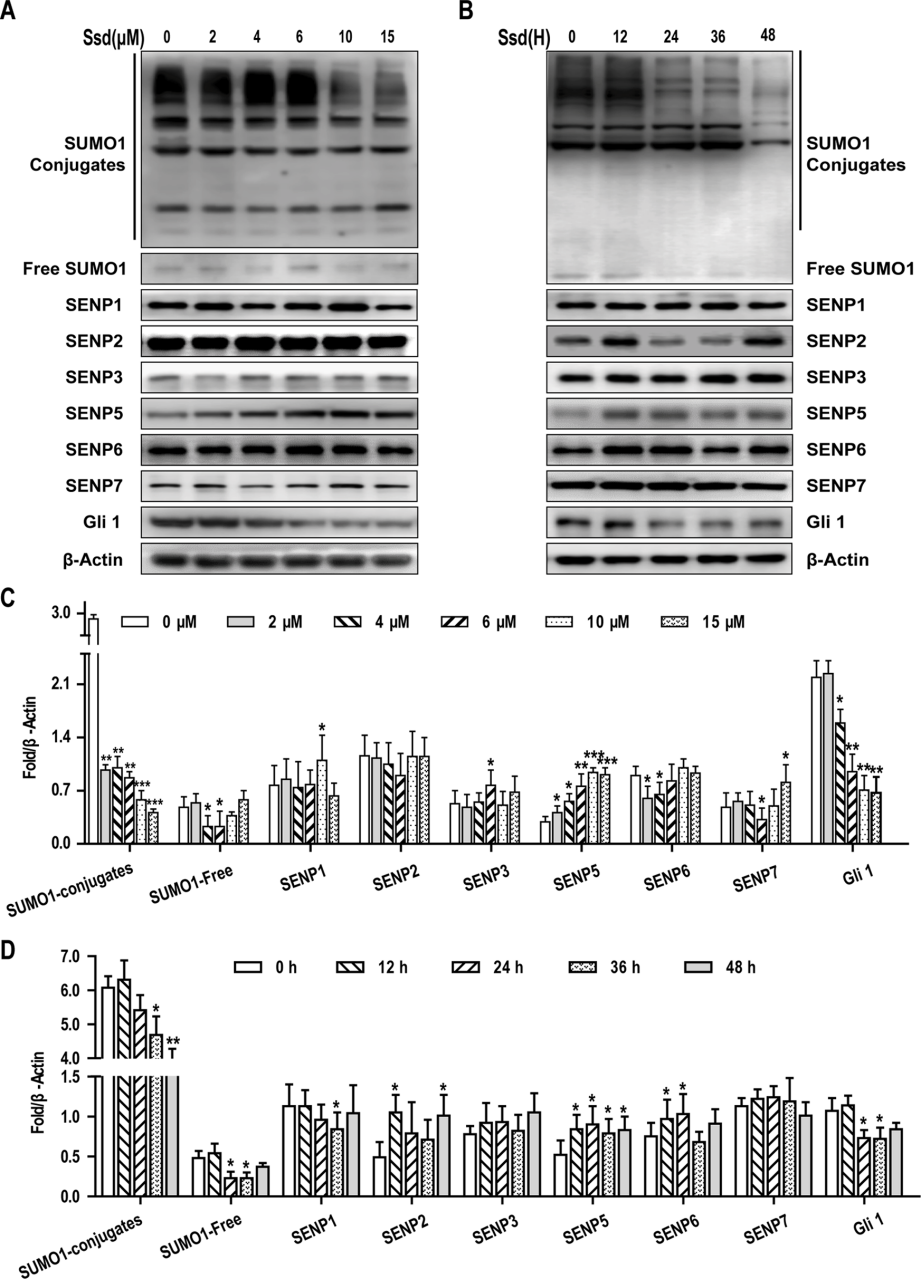
Saikosaponin-d (Ssd) may inhibit the SUMOylation of Gli1 by specifically upregulating the expression of SENP5. **(A)** Concentration-dependent effects of Ssd treatment on SUMO1, SENP1–3, SENP5–7, and Gli1 protein expression in Hep3B cells, as determined by western blotting. Cells were treated with the indicated concentrations of Ssd for 48 h. **(B)** Time-dependent effects of Ssd treatment (6 µM) on the same proteins examined in **(A)**. **(C**, **D)** Quantitative analysis of western blotting data presented in **(A** and **B)**. Protein expression data are normalized to the β-actin control and are expressed as means ± SD (n = 3); *P < 0.05 and **P < 0.01 when compared with control (Student’s t test).

In this study, we found that SUMOylation was involved in the activation of the SHh pathway, and that SHh signaling promoted EMT and chemosensitivity in HCC cells. Therefore, targeting of the SUMO pathway may have a therapeutic benefit in the treatment of this disease. In our initial experiments, we treated Hep3B cells with eight biologically active components of traditional Chinese medicine that have been reported to have a significant inhibitory effect on HCC cells. Among these, Ssd was found to significantly reduce the expression of SUMO1 in these components, as determined by western blotting (**Supplementary Figure 1**). Given that Ssd suppresses SUMO1 expression in HCC cells, we next wished to determine whether Ssd exerts its inhibitory effects on Hep3B cells through modulation of the SUMO pathway. Consequently, we next examined the concentration and time-dependent effects of Ssd treatment on the expression of SUMO1 and SENP family proteins, key regulators of SUMO processing, in Hep3B cells. Western blotting analysis demonstrated that Ssd markedly downregulated SUMO1 expression while specifically upregulating SENP5 expression in a time- and dose-dependent manner. Meanwhile, we found that Ssd had no significant effect on SUMO2/3 at different concentrations (**Supplementary Figure 2**). In addition, Ssd significantly downregulated the level of Gli1 expression (**Figures 5A–D**). Taken together, these results indicate that Ssd may inhibit the SUMOylation of Gli1 by specifically upregulating the expression of SENP5.

### Ssd Inhibits HCC Tumor Growth and Promotes Sensitivity to Hsvtk/GCV *In Vivo*

**Figure 6:**
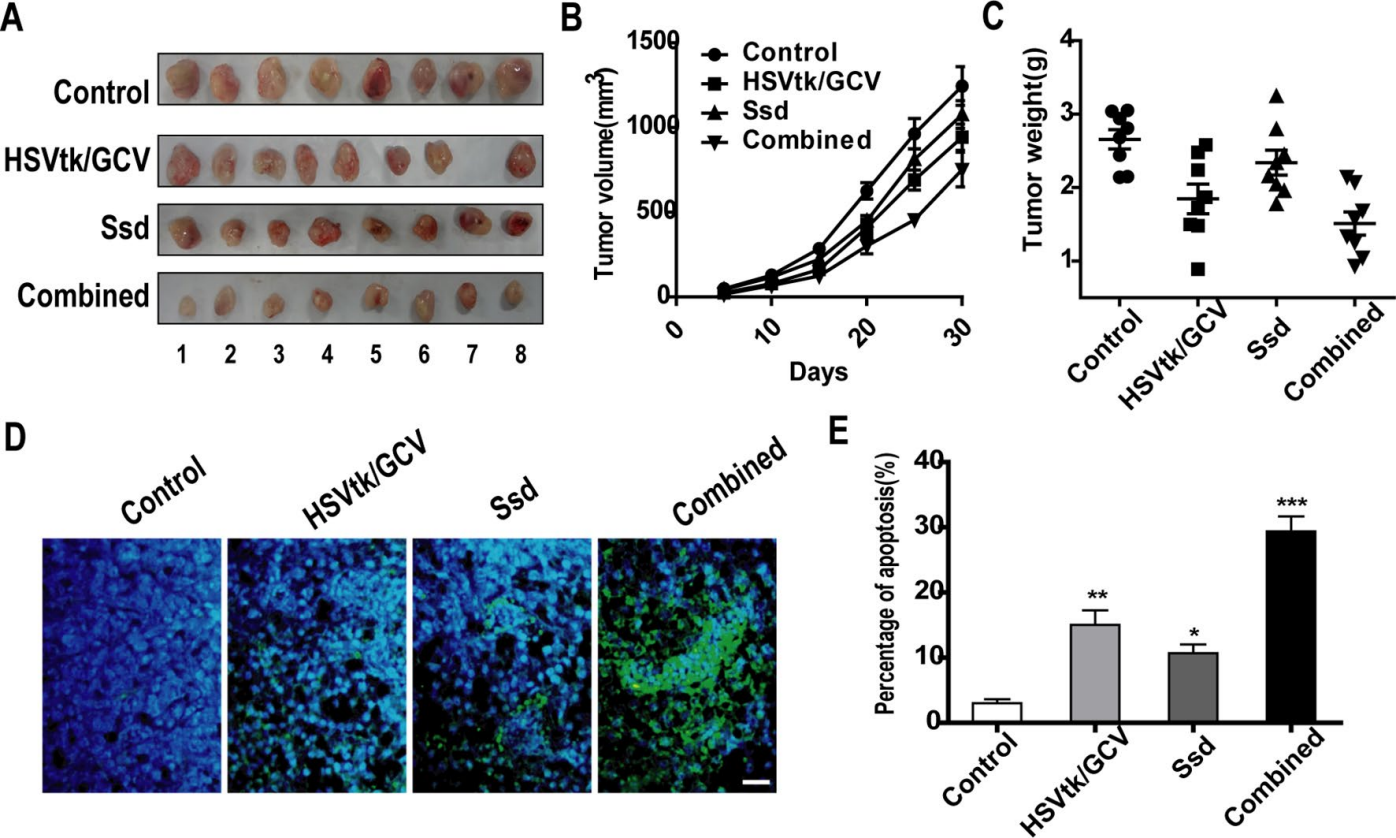
Saikosaponin-d (Ssd) inhibits tumor growth and increases the sensitivity of hepatocellular carcinoma (HCC) cells to herpes simplex virus thymidine kinase/ganciclovir (HSVtk/GCV) *in vivo*. BALB/c nude mice were inoculated with HSVtk/Hep3B cells and subsequently treated with vehicle, Ssd, GCV, or a combination of Ssd and GCV. **(A)** Subcutaneous tumors were dissected from nude mice and photographed. **(B)** Tumor volumes were measured every 3 days over a 30-day period. **(C)** Tumors were weighted immediately after dissection from nude mice. **(D)** Apoptosis in tumor xenograft sections was detected by TUNEL staining (scale bar, 25 µm). **(E)** Quantification of apoptosis in tumor sections. Data are expressed as means ± SD (n = 3); *P < 0.05, **P < 0.01, and ***P < 0.001 when compared with the control (Student’s t test).

To verify the tumor-suppressive and chemosensitizing effects of Ssd *in vivo*, a tumor xenograft model was established by injecting HSVtk/Hep3B cells subcutaneously into immunodeficient mice. As shown in **Figures 6A–C**, tumor volume and weight were markedly decreased in the HSVtk/GCV tumor group (where animals were also subjected to GCV intraperitoneal injection) when compared with the control tumor group. A similar reduction in tumor volume and weight was also observed for the Ssd tumor group. However, the volumes and weights of HSVtk/Hep3B tumors treated with a combination of Ssd and GCV were much lower than those of tumors in the individual treatment groups. In addition, *in situ* detection of apoptosis demonstrated that the HSVtk/GCV system combined with Ssd treatment significantly elevated the level of apoptosis within the tumor (**Figures 6D, E**).

In summary, Ssd significantly inhibited the proliferation of HCC cells and increases their chemosensitivity *in vivo*.

## Discussion

Chemosensitivity has always been the focus of clinical discussion, and drug resistance is one of the important reasons for tumor failure and disease progression. Here, our team first discovered the following points: (1) hypoxia greatly enhanced the malignant phenotype of Hep3B cells, induced activation of the SHh pathway, promoted EMT and reduced resistance to HSVtk/GCV. (2) Ssd can reverse the adverse consequences caused by hypoxia, providing a new hope for the clinical diagnosis and treatment of liver cancer. (3) Ssd especially activated the expression of SENP5, a specific deSUMOylation enzyme, while inhibiting the expression of SUMO1 and Gli1, which may be related to the molecular mechanism on the Ssd inhibiting the growth of Hep3B and increasing the sensitivity to chemotherapy.

Hypoxia is a common characteristic of rapidly growing malignant tumors and is known to promote cancer progression. Studies have shown that hypoxia has a negative impact on the efficacy of both radiotherapy and chemotherapy in various cancer types, including mammary carcinoma (Gilkes and Semenza, 2013), head and neck carcinoma (Clavo et al., 2017), and uterine cervix carcinoma (Durand and Aquino-Parsons, 2006). Therefore, hypoxia is considered to be a potential therapeutic target for various solid tumors to help alleviate tumor resistance to these common forms of therapy.

Ssd, a major biologically active triterpenoid saponin, has previously been reported to inhibit tumor growth and enhance radiosensitivity in hepatoma cells under hypoxic conditions (Wang et al., 2014). Wang et al. (2013) Wang et al. (2014) showed that Ssd enhances radiosensitivity of HCC cells, which may contribute to its effect on the G0/G1 and G2/M checkpoints of the cell cycle or inhibiting hypoxia-inducible factor-1α. Inspired by their findings, we regarded hypoxic microenvironment as a research condition in this experiment, and the results that Ssd inhibits the malignant phenotype and sensitizes HSVtk/Hep3B cells to GCV are basically consistent with those of predecessors (He et al., 2014).

However, there are few studies on the mechanisms regulating chemosensitivity in liver cancer. The SHh pathway is considered to play a crucial role in tumorigenesis and cancer (Ciepla et al., 2014). The three major components of the pathway are the SHh signal peptide, the transmembrane receptor complex PTCH/SMO, and the zinc finger protein transcription factor GLI. The GLI family has three homologues, namely, Gli1, Gli2, and Gli3. Gli1 and Gli2 function predominantly as transcriptional activators, whereas Gli3 is a major downstream inhibitor of SHh signaling (Zhang et al., 2019). Aberrant GLI signaling has been observed in a number of cancers and is associated with poor prognosis, driving processes such as tumorigenesis, invasion, and metastasis (Rovida and Stecca, 2015). Subsequently, we performed a systematic analysis of GLI protein expression in tumor tissues and matched adjacent normal tissues from HCC patients. Our analysis revealed that all GLI family proteins are highly expressed in HCC tissues and that, when compared with adjacent normal tissues, these proteins are predominantly localized to the cytoplasm.

Accumulating evidence supports a role for the SHh signaling pathway in the promotion of EMT (Islam et al., 2016; Zhang et al., 2018). Hypoxia within the local microenvironment can also impact tumor malignancy, promote the proliferation, and induce EMT and antitumor drug resistance (Qiu et al., 2017). So, we treated Hep3B cells with hypoxic conditions and found that hypoxia activated the SHh pathway to promote EMT, invasion, and chemosensitivity in HCC cells.

SUMOylation is a reversible protein posttranslational modification that alters target protein function and is implicated in a variety of cellular processes, including the regulation of gene transcription, DNA replication and repair, and protein transport, localization, stability, and interaction. Abnormal SUMOylation have been implicated in a variety of disorders, including cancer (Henley et al., 2014; Hendriks and Vertegaal, 2016). Fausto et al. have shown that GLI family proteins can be regulated by SUMOylation (Cox et al., 2010; Han et al., 2012; Ma et al., 2016; Hoard et al., 2018). However, the mechanism by which hypoxia affects the SUMO modification of these proteins in HCC remains unclear. The potential for Ssd to enhance the chemosensitivity of HCC by altering the SUMOylation of GLI has yet to be investigated.

Then, we found that expressions of SUMO1 and Ubc9 were increased in HCC and that these proteins exhibited the same subcellular localization as GLI family proteins. We also examined the role of SUMOylation in the hypoxia-dependent activation of SHh signaling and have demonstrated that SUMO is involved in the development of HCC. We have shown that Ssd can significantly inhibit the expression of SUMO1 but not SUMO2/3 (**Supplementary Figure 2**), thereby promoting GLI activation, which is important for the promotion of liver cancer. We therefore speculated that the inhibitory effects of Ssd on HCC may involve the SUMOylation of multiple proteins. Interestingly, we found that Ssd could specifically activate SENP5, a deSUMOylation enzyme, in a time- and dose-dependent manner, thereby may be inhibit the SUMOylation of Gli1 in HCC cells.

Finally, although we have shown that Ssd inhibits the malignant phenotype in HCC cells and increases chemosensitivity, which may be related to specifically upregulating the expression of SENP5 and then inhibiting the SUMOylation of Gli1. The precise mechanism of Ssd on HCC cells remains unclear, and we also need to further study this in the future.

## Data Availability

The raw data supporting the conclusions of this manuscript will be made available by the authors, without undue reservation, to any qualified researcher.

## Ethics Statement

The studies involving human participants were reviewed and approved by Ethics Committee of the Fifth Central Hospital of Tianjin. The patients/participants provided their written informed consent to participate in this study. The animal study was reviewed and approved by Ethics Committee of the Fifth Central Hospital of Tianjin.

## Author Contributions

W-HW designed the experiments. C-YZ, Z-MJ, X-FM and YL performed the experiments and collected data. C-YZ, Z-MJ, X-ZL and L-LL analyzed and interpreted the data. C-YZ, Z-MJ drafted the manuscript. C-YZ, TW and W-HW revised the paper critically for important intellectual content. T-W and W-HW agreed to be accountable for all aspects of the work in ensuring that questions related to the accuracy or integrity of any part of the work are appropriately investigated and resolved. All authors read and gave final approval of the version to be published.

## Funding

This study was supported by grant from National Natural Science Foundation of China (Grant No. 31671246, 81471175 and 81501073), Tianjin Natural Science Foundation of China (Grant No. 18JCQNJC12800), and Binhai Health and Family Planning Commission Science and Technology Projects (Grant No. 2016BWKY005).

## Conflict of Interest Statement

The authors declare that the research was conducted in the absence of any commercial or financial relationships that could be construed as a potential conflict of interest.
