# Supplementary material for: Saikosaponin-d Inhibits the Hepatoma Cells and Enhances Chemosensitivity Through SENP5-Dependent Inhibition of Gli1 SUMOylation Under Hypoxia
Source: Front Pharmacol. 2019 Sep 20;10:1039. doi: 10.3389/fphar.2019.01039 (PMC6764240; doi:10.3389/fphar.2019.01039)
Supplement: Supplementary file 1 [file Presentation_1.pdf]

Saikosaponin-d inhibits the hepatoma cells and enhances chemosensitivity through SENP5-dependent inhibition of Gli1 SUMOylation under hypoxia

Chun-Yan Zhang[1,2†], Zhong-Min Jiang[3†], Xiao-Fang Ma[4], Yue Li[1], Xiao-Zhi Liu[4], Li-Li Li[5,6,7], Wen-Han Wu[8,9]* and Tao Wang[2*]

[1]Department of Pharmacy, Tianjin Binhai New Area Hospital of Traditional Chinese Medicine, Tianjin 300450, China,

[2]Tianjin State Key Laboratory of Modern Chinese Medicine, Tianjin University of Traditional Chinese Medicine, Tianjin 300193, China,

[3]Department of Pathology, [4]Central Laboratory, [9]Department of General Surgery, The Fifth Central Hospital of Tianjin, Tianjin 300450, China,

[5]Department of Bone and Soft Tissue Tumors, Tianjin Medical University Cancer Institute and Hospital, Tianjin 300060, China,

[6]National Clinical Research Center for Cancer, Tianjin Medical University Cancer Institute and Hospital, Tianjin 300060, China,

[7]Key Laboratory of Cancer Prevention and Therapy, Tianjin Medical University Cancer Institute and Hospital, Tianjin 300060, China,

[8]Department of General Surgery, Peking University First Hospital, Beijing 100034, China

The chemosensitivity is one of the key factors affecting the therapeutic effect of cancer, but the clinical application of corresponding drugs is rare. Hypoxia, a common feature of many solid tumors, including hepatocellular carcinoma (HCC), has been associated with resistance to chemotherapy, in part through the activation of the SHh pathway. Hypoxia has also been associated with the increased SUMOylation of multiple proteins, including GLI family proteins, which are key mediators of SHh signaling, and has become a promising target to develop drug-resistant drugs for cancer treatment. However, there are few target drugs to abrogate the chemotherapy resistant. Saikosaponin-d (Ssd), one of the main bioactive components of Radix Bupleuri, has been reported to exert multiple biological effects, including anti-cancer activity. Here, we first found that Ssd inhibits the malignant phenotype of HCC cells while increasing their sensitivity to the herpes simplex virus thymidine kinase/ganciclovir (HSVtk/GCV)

drug system under hypoxia in vitro and in vivo. Furthermore, we had explored that GLI family activation and extensive protein SUMOylation were characteristics of HCC cells, and hypoxia could activate the SHh pathway, and promote epithelial-mesenchymal transition (EMT), invasion and chemosensitivity in HCC cells; and SUMOylation is required for hypoxia-dependent activation of GLI proteins. Finally, we found that Ssd could reverse the effects promoted by hypoxia, specifically active sentrin/small ubiquitin-like modifier (SUMO)-specific protease 5 (SENP5), a SUMO-specific protease, in a time- and dose-dependent manner, while inhibit the expression of SUMO1 and GLI proteins. Together, these findings confirm the important role of Ssd in the chemoresistance of liver cancer, provide some data support for further understanding the molecular mechanism of Ssd inhibition of malignant transformation of HCC cells, and provide a new perspective for the application of traditional Chinese medicine in the chemical resistance of liver cancer.

* **Correspondence:**
Corresponding Author

Dr. Wen-Han Wu, Peking University First Hospital, Beijing, China,
wuwenhan88@126.com

Mx. Tao Wang, Tianjin University of Traditional Chinese Medicine, Tianjin, China,
wangtao@tjutcm.edu.cn, wangtao@tjutcm.edu.cn

† Contributed equally.

Keywords: Saikosaponin-d; hepatocellular carcinoma; HSVtk/GCV; Gli1; SUMOylation; SENP5; Hep3B cells; hypoxia

1 Supplementary Figures

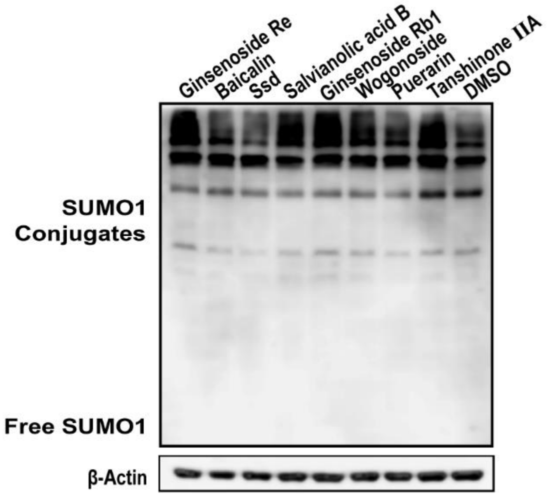

Supplementary Figure 1. The effect of known bioactive components of traditional Chinese medicine on SUMO1 expression in Hep 3B cells. Note the suppressive effects of Ssd treatment on SUMO1 expression.

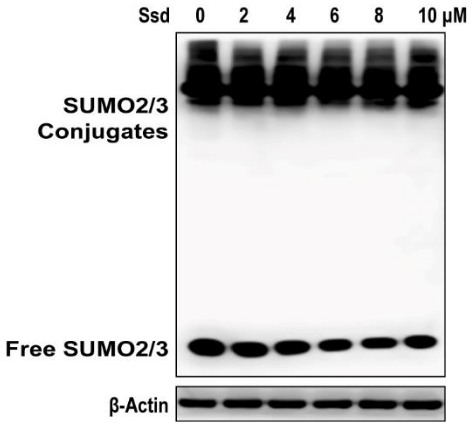

Supplementary Figure 2. Evaluation of the concentration-dependent effects of Ssd on SUMO2/3 expression in Hep 3B cells. Note that Ssd had no significant effect on SUMO2/3 expression at all of the concentrations tested.
